# High-resolution multimodal flexible coherent Raman endoscope

**DOI:** 10.1038/s41377-018-0003-3

**Published:** 2018-05-30

**Authors:** Alberto Lombardini, Vasyl Mytskaniuk, Siddharth Sivankutty, Esben Ravn Andresen, Xueqin Chen, Jérôme Wenger, Marc Fabert, Nicolas Joly, Frédéric Louradour, Alexandre Kudlinski, Hervé Rigneault

**Affiliations:** 1 Aix-Marseille Univ, CNRS, Centrale Marseille, Institut Fresnel, Marseille, France; 2 Laboratoire de Physique des Lasers Atomes et Molécules, UMR 8523, CNRS, Université Lille, 59000 Lille, France; 3 CNRS, XLIM, UMR 7252, Université de Limoges, 87060 Limoges, France; 4 Department of Physics, Max Planck Institute for the Science of Light, University of Erlangen Nuremberg, 91058 Erlangen, Germany

## Abstract

Coherent Raman scattering microscopy is a fast, label-free, and chemically specific imaging technique that shows high potential for future in vivo optical histology. However, the imaging depth in tissues is limited to the sub-millimeter range because of absorption and scattering. Realization of coherent Raman imaging using a fiber endoscope system is a crucial step towards imaging deep inside living tissues and providing information that is inaccessible with current microscopy tools. Until now, the development of coherent Raman endoscopy has been hampered by several issues, mainly related to the fiber delivery of the excitation pulses and signal collection. Here, we present a flexible, compact, coherent Raman, and multimodal nonlinear endoscope (4.2 mm outer diameter, 71 mm rigid length) based on a resonantly scanned hollow-core Kagomé-lattice double-clad fiber. The fiber design enables distortion-less, background-free delivery of femtosecond excitation pulses and back-collection of nonlinear signals through the same fiber. Sub-micrometer spatial resolution over a large field of view is obtained by combination of a miniature objective lens with a silica microsphere lens inserted into the fiber core. We demonstrate high-resolution, high-contrast coherent anti-Stokes Raman scattering, and second harmonic generation endoscopic imaging of biological tissues over a field of view of 320 µm at a rate of 0.8 frames per second. These results pave the way for intraoperative label-free imaging applied to real-time histopathology diagnosis and surgery guidance.

## Endoscopy: Bringing coherent Raman microscopy into the body

A powerful technique called coherent Raman scattering microscopy could be used to visualize and study tissue inside the body thanks to a new light-transmitting fiber technology. Coherent Raman scattering generates images based on the interaction of light with  molecules. It opens a window into cells and tissues that reveals information unavailable with simple illumination by light. Applying the technique deep within the body has proved difficult due to technical problems in delivering the necessary ultra-short pulses of light, and collecting the signals that are scattered back. Researchers in France and Germany, led by Hervé Rigneault at the Fresnel Institute in France, combined several innovations in fiber technology to bring high-resolution coherent Raman imaging into previously hidden locations. Their flexible endoscope literally shines new light to assist the diagnosis and surgical treatment of cancer and other diseases.

## Introduction

Identification of cancer tumors is generally carried out ex vivo by histological inspection of tissue biopsies. This process requires several steps, such as tissue resection, sectioning, and staining, and is therefore time-consuming and labor-intensive. Realization of real-time label-free in vivo endoscopic imaging would represent a major breakthrough in histology. By enabling direct visualization of cancerous cells and tissues inside a patient’s body, such an endoscope system would revolutionize current approaches for cancer diagnosis and intraoperative surgery decision-making.

As a significant step towards achieving this goal, coherent anti-Stokes Raman scattering (CARS), and stimulated Raman scattering (SRS) are fast, label-free, and chemically specific imaging techniques^1,2^ that show high potential for in vivo intraoperative histopathology^3–5^. However, despite the capacity of CARS and SRS to provide high-resolution microscopy images of unprocessed samples, these techniques cannot be used to image deeper than a few hundred micrometers inside tissues as they are limited by optical absorption and scattering^6^. Moreover, CARS and SRS are implemented using unwieldy microscope setups on top of optical tables, and therefore, miniaturization and portability remain challenging issues for clinical applications.

Performing CARS and/or SRS imaging via a fiber endoscope system is a crucial step towards imaging deep inside tissues and providing chemical imaging with a spatial resolution that is inaccessible using current fibered Raman probes^7,8^. Despite significant efforts over the last decade^9–15^, major technical challenges for pulsed laser delivery and signal collection have hindered the development of coherent Raman endoscopy. CARS/SRS endoscopy is more demanding than two-photon excited fluorescence (TPEF) or second harmonic generation (SHG)^16–18^ as two spatially and temporally overlapping excitation beams are required. When these two pulses co-propagate in a fiber, a strong parasitic background arises due to the occurrence of nonlinear four wave mixing (FWM) in the fiber core^9^. Moreover, group velocity dispersion (GVD) and a variety of nonlinear effects can affect pulse propagation in fibers, leading to pulse temporal and spectral broadening as well as a loss of peak intensity^19^. For CARS and SRS, GVD and pulse propagation issues have to be compensated at two different wavelengths, which further complicate the challenge. To address these problems, previous studies have used different fibers for excitation and collection^9,13,15^ or dedicated fibers with a large mode area^11^ or polarization control^10^. However, the complexity introduced by these designs leads to bulky endoscope heads and/or a loss in optical resolution and contrast, which are not compatible with in vivo histopathology applications.

In this article, we present a compact flexible fiber-optic scanning endoscope dedicated to high-resolution coherent Raman imaging deep inside tissues. Our specific design combines several key innovations: (1) a broadband hollow-core (HC) fiber with a Kagomé lattice to guide the two co-propagating laser beams with negligible FWM background, GVD, and pulse distortion. (2) Double cladding around the HC fiber with a high numerical aperture which provides efficient signal back-collection in scattering samples. (3) A silica microsphere inserted into the output facet of the HC fiber core solves the issue related to the large mode surface of the HC fiber and enables a submicron spatial resolution for CARS imaging. (4) A miniature resonant piezo scanner combined with a distal miniature objective provides an electrically tunable field of view (FoV) of a few hundreds of µm while preserving the compactness of the endoscope distal head (4.2 mm outer diameter, 71 mm rigid length). This unique combination of features enables high-contrast and high-resolution multimodal nonlinear endoscopy of unstained tissues. We perform CARS, SHG, and TPEF endoscopy imaging with submicron spatial resolution over a FoV of up to 320 µm at a rate of 0.8 frames per second. By solving most of the issues raised in nonlinear endoscopy, our dedicated design opens a major route towards clinical application of deep in vivo CARS imaging for real-time histopathology diagnosis.

## Materials and methods

### Endoscope system overview

**Fig. 1:**
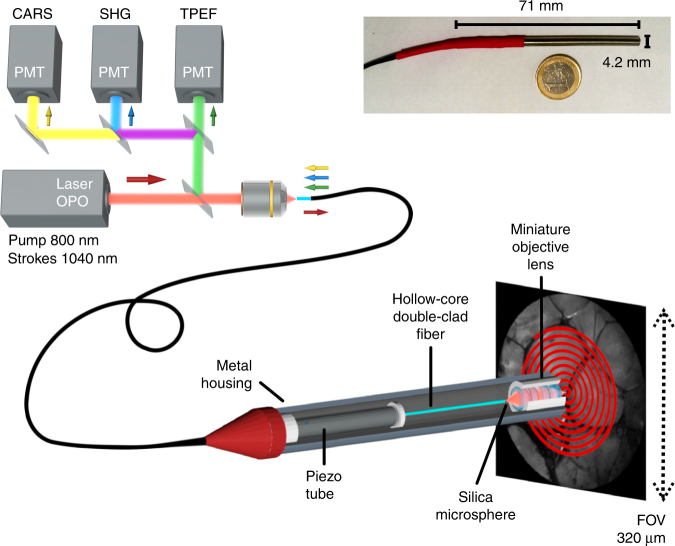
Coherent Raman endoscope overview. A tunable femtosecond laser provides two synchronized pulse trains that are injected into the hollow-core fiber probe to perform CARS, SHG, and TPEF endoscopy. The light emitted by the sample is collected by and back-propagates through the same fiber (1 m long) before detection by large area photomultiplier detectors. The inset picture shows the endoscope probe head inserted into its stainless steel housing

Our multimodal nonlinear endoscope setup is shown in Fig. 1. A tunable multi-wavelength laser system (Discovery, Coherent) provides two synchronized fs ultra-short 80 MHz pulse trains for the pump (800 nm—100 fs) and Stokes (1040 nm—160 fs) beams. These wavelengths are suitable to address carbon hydrogen (C–H) bonds (~2885 cm^−1^) with CARS. The two co-propagating laser pulses are temporally overlapped with a mechanical delay line and injected into the same fiber core. After propagating through 1 m of fiber, the laser spot at the fiber distal facet is further re-imaged onto the sample through a custom designed miniature objective based on four commercial achromatic doublets (Fig. S1). The magnification of the miniature objective is 0.63, with a numerical aperture of 0.3 on the fiber side and 0.45 on the sample side. The objective working distance is 0.6 mm in air and is sufficiently long for tissue imaging applications.

For image scanning, the distal end of the fiber is attached to a four-quartered piezo tube (PT230.94, Physik Instrument, Karlsruhe, Germany—diameter 3.2 mm), with a free standing length of 23 mm. The excitation spot is scanned over the sample by resonant driving of the piezo tube following an expanding spiral pattern (Fig. S2). The FoV of the endoscope distal probe is a circle with a maximum diameter of 320 µm, which results from the de-magnification of the 510 µm fiber scan (at 30 V driving voltage) through the miniature objective lens. The whole endoscope distal head is integrated into a biocompatible stainless steel tube (4.2 mm diameter—length 71.2 mm), which ensures portability (Fig. 1, inset picture).

The optical signal generated in the sample is back-collected through the same objective and fiber. After propagation through the fiber, the signal is spectrally filtered by a set of dichroic mirrors and bandpass filters before detection by photomultiplier units (H7421-40, Hamamatsu Photonics, Japan). CARS, SHG and TPEF images are built and displayed in real time using custom LabVIEW software based on an open-loop image reconstruction algorithm (Figs. S3, S4). The details of the experimental setup can be found in the supplementary information.

### Double-clad hollow-core fiber probe

**Fig. 2:**
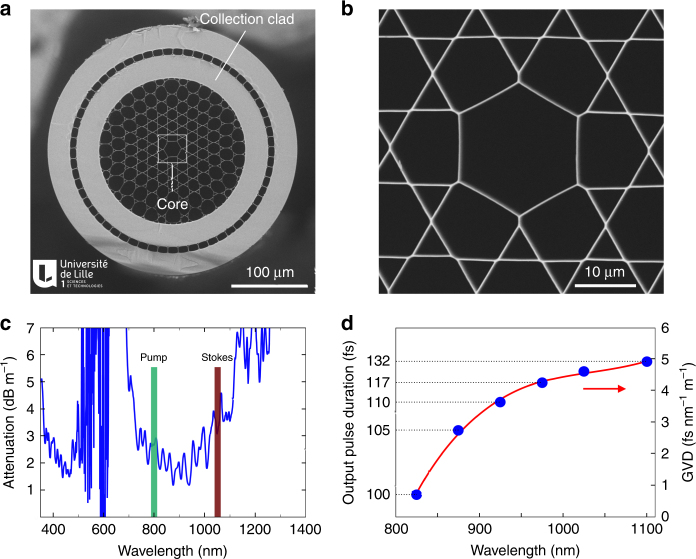
Hollow-core photonic crystal fiber for multimodal nonlinear endoscopy. **a** Electron microscope image of the hollow-core (HC) fiber featuring a Kagomé lattice together with a double cladding (DC) separated by air holes. The excitation pulses propagate through the HC, whereas the nonlinear signal is collected and transmitted through the DC. **b** Close-up view of the fiber core area. **c** Attenuation of the hollow fiber core; the wavelengths used for the pump and Stokes beams are highlighted. **d** Expected pulse duration after propagation over 1 m of fiber, for initially transform-limited 100 fs pulses. The output pulse duration is computed using the  experimental GVD values (blue dots—right scale), which are  obtained from the measurement of the group delay versus wavelength. The expected duration for a 150 fs input pulse centered at 1040 nm (the Stokes pulse) is 157 fs after 1 m of fiber. The red line is a third-order polynomial fit to the GVD data

One of the key elements of our nonlinear multimodal endoscope is the hollow-core (HC) fiber^20^ (Fig. 2a, b), which combines a Kagomé-lattice HC for distortion-less femtosecond pulse delivery and a high numerical aperture all silica double cladding (DC) for nonlinear signal collection. The fiber has an outer diameter of 327 µm and a core diameter of 20 µm wherein light propagates in a single transverse mode. Light confinement inside the Kagomé core is not the result of a photonic band gap, but is due to a mechanism based on inhibited coupling between the cladding modes and guided core modes^21^. The transmission window extends from 700 to 1100 nm with propagation losses below 5 dB m^−1^ (Fig. 2c), which covers the 2-photon excitation spectra of most fluorescent labels and is large enough to accommodate both the pump and Stokes beams for CARS. Importantly, the GVD is very small and remains below 5 fs nm^−1^ m^−1^ over the full transmission window; hence, the temporal broadening of a ~150 fs pulse traveling over the 1 m fiber length is only a few fs and can thus be fully neglected (Fig. 2d). In addition, we observe negligible spectral changes following signal propagation through the fiber, even for powers of up to 60 mW (Fig. S5). Altogether, these results show that the hollow-core fiber is suitable for delivering high-power femtosecond laser pulses with negligible distortion over a broad spectral window.

The second main challenge in fiber design for coherent Raman imaging is the parasitic background due to nonlinear four wave mixing (FWM) occurring in the fiber core. Figure S6 shows that the HC of our fiber design can heavily suppress the parasitic FWM background, enabling noise-free CARS detection. This is in sharp contrast with most solid-core fibers, which necessitate FWM filtering^13,15^.

The third challenge for CARS endoscopy is the ability to collect a nonlinear signal even in the presence of a scattering sample. To solve this issue, our fiber design features a second annular silica cladding dedicated to the collection of the generated nonlinear signals (Fig. 2a). Broadband and multimode guidance in this silica double cladding (DC) is obtained by adding a low index ring of air holes (air clad) outside the Kagomé structure. The inner and outer diameter for this cladding is 190 µm and 250 µm, respectively, providing a total collection surface of ~21,000 µm^2^ with a large numerical aperture of ~0.5. This DC is necessary to collect the CARS, SHG, and TPEF, the wavelengths of which lie outside the hollow-core transmission window. The high numerical aperture increases the endoscope collection efficiency when working with scattering samples such as biological tissues (Fig. S8).

### Microsphere lens focusing for a hollow-core fiber probe

**Fig. 3:**
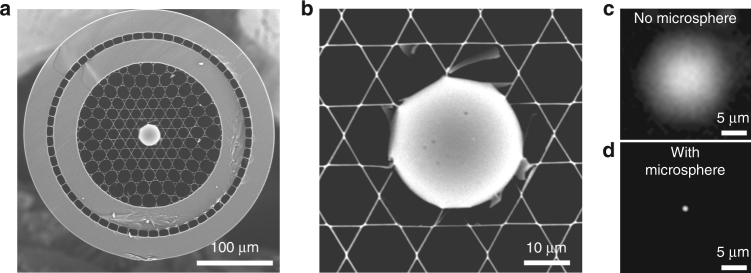
Microsphere lens inserted into the hollow fiber core provides a submicron focus spot required for imaging. **a** Scanning electron microscope image of the Kagomé HC fiber with a 30 µm silica microsphere inserted and sealed into the fiber core. **b** Close-up view of the microsphere inserted into the hollow core. **c** In the absence of the microsphere, the HC mode diameter is 15 µm, which is inappropriate for high-resolution nonlinear imaging. **d** The microsphere acts as a ball lens and focuses the light exiting the fiber core into a ~1 µm diameter spot

So far, we have shown that Kagomé-lattice hollow-core fibers can have major advantages for nonlinear endoscopy. However, such fibers also feature one significant drawback related to their large guided mode diameter, which is inappropriate for high-resolution nonlinear imaging. To overcome this issue, we use a 30 µm silica microsphere inserted into the hollow fiber core (Fig. 3a, b). The microsphere acts as a ball lens to strongly focus the ~15 µm diameter guided mode into a ~1 µm focus spot^22,23^ (Fig. 3c, d). This focus spot is then imaged onto the sample plane by the miniature objective, which acts as a relay lens (Fig. 1). This is the key to achieve submicron image resolution and a large FoV, as we will show hereafter. To maintain contact between microsphere and fiber at a high piezo scanning speed, the microsphere was permanently sealed into the fiber by using a CO_2_ laser splicer. The high damage threshold and low absorption of silica enabled the delivery of excitation powers higher than 100 mW with no visible impact on device performance. The microsphere generates a FWM signal when the pump and Stokes pulses temporally overlap at the fiber exit. However, the GVD of the miniature objective induces an additional delay between the beams. When performing CARS imaging, the FWM generated by the bead is negligible because the pulses temporally overlap on the sample but not at the fiber exit (Fig. S6).

## Results and discussion

**Fig. 4:**
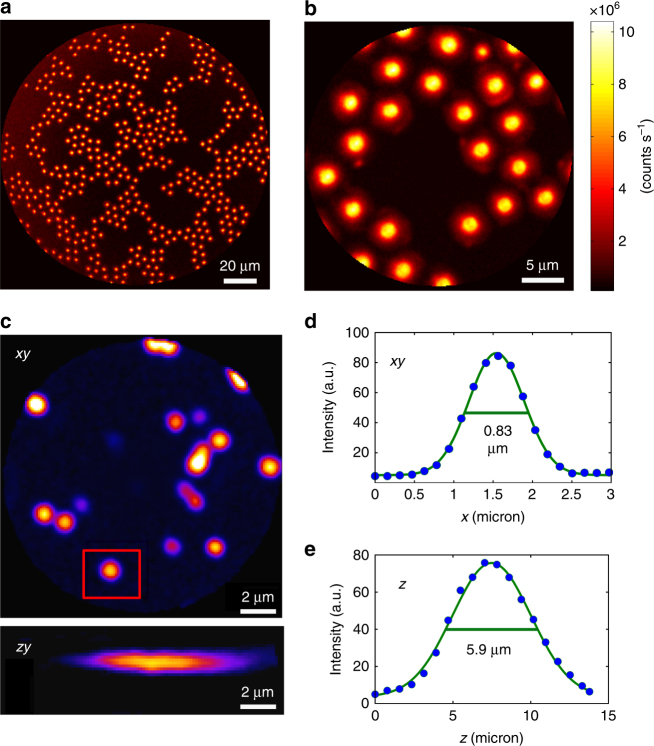
High-contrast submicron image resolution. **a**, **b** CARS images of 5-µm diameter polystyrene beads deposited onto a glass coverslip with two different FoVs: 155 µm (**a**) and 25 µm (**b**) corresponding to 7.5 V and 1.5 V pk-pk driving voltages, respectively. Note the vanishing background between the beads; powers: 10 mW (pump) and 5 mW (Stokes); excitation wavelengths: 800 nm (pump) and 1040 nm (Stokes). **c** Lateral (*x*,*y*) and axial (*x*,*z*) TPEF images (forward detected) of 200-nm-diameter fluorescent nanoparticles (excitation: 800 nm, 10 mW; detection: 500–600 nm). The nanoparticle highlighted in the red rectangle is used to obtain the image cross-cuts (**d**, **e**), which are used to deduce the lateral and axial two-photon PSFs. The values shown on the graph indicate the full width at half maximum (FWHM)

We first demonstrate the imaging capabilities of our endoscope system using 5 µm polystyrene beads deposited onto a glass coverslip. Figure 4a and b shows CARS images acquired using two different driving voltages for the piezo scanner, which provide different FoVs of 155 µm and 25 µm, respectively. A strong CARS signal is generated by each bead in response to the excitation beams. The background between the beads is remarkably dark, which demonstrates CARS imaging free of a fiber FWM background.

To measure the lateral and axial 2-photon resolution of our endoscope probe, we record the TPEF image of 200 nm fluorescent nanoparticles excited by a laser pulse at 800 nm (Fig. 4c). The image of an individual nanoparticle provides the point spread function (PSF) of the endoscope. We achieve a 2-photon lateral resolution (PSF full width at half maximum) of 0.83 µm (Fig. 4d), whereas the axial resolution is 5.9 µm (Fig. 4e). Both values are in good agreement with the expected NA = 0.45 for focusing onto the sample. CARS PSF is more difficult to measure using small beads because of the non-resonant FWM background from the substrate. However, we estimated the PSF to be approximately equal to 1 µm in the lateral direction and 7.7 µm in the axial direction (Fig. S8). In addition, we checked that the PSF is not significantly altered when the FoV is increased up to 350 µm (Fig. S9). This leads to a remarkable flatness of field as illustrated on the USAF test chart (Fig. S10).

**Fig. 5:**
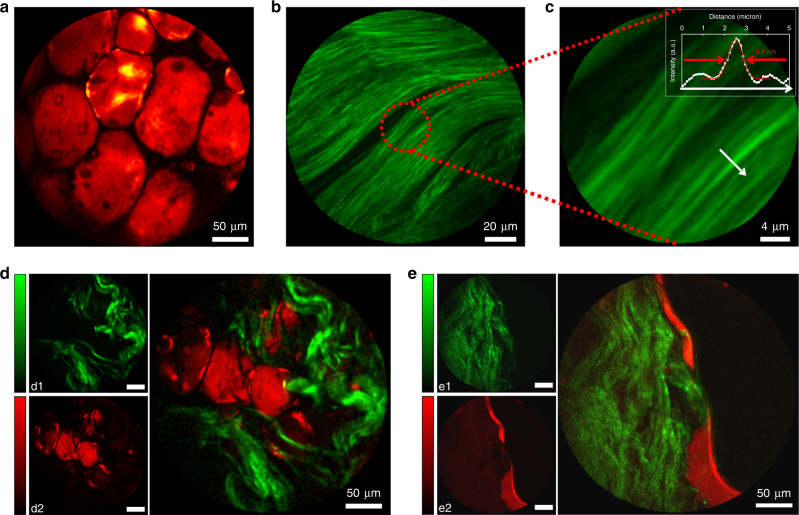
Multimodal nonlinear endoscopy in biological tissues. **a** CARS image of fresh fatty tissues obtained from human colon. **b** SHG image of rat tail tendons featuring collagen fibers. **c** Close-up view of **b** confirming the 0.8 µm lateral resolution for nonlinear imaging. **d**, **e** Multimodal SHG (green, d1, e1) and CARS (red, d2, e2) images of fresh human colon tissues taken 50 µm below the sample surface. The large images are overlaps of the SHG and CARS images. SHG images required higher powers and were acquired immediately after taking the CARS images. Laser powers incident on the sample: CARS pump 20 mW, Stokes 10 mW; SHG 60 mW (45 mW for images **b** and **c**). Excitation wavelengths: 800 nm (pump CARS, SHG) and 1040 nm (Stokes). All images were averaged over five acquisitions for a total acquisition time of 6.5 s, except **b** and **c** that were averaged 10 times (13 s). The color scale in (counts per second) is provided on Fig. S14

We now move to label-free tissue samples to demonstrate the capability for CARS and SHG multimodal nonlinear endoscopy imaging. Figure 5a shows a CARS image obtained for a fresh human colon fatty tissue sample, where the high lipid content provides CARS contrast through the C–H stretch vibrations. The tissue was sandwiched in water between two glass coverslips inside a 1 mm-thick spacer and vertically mounted in front of the endoscope probe. Again as in Fig. 4a and b, we find an excellent CARS contrast together with a high spatial resolution. We also demonstrate that a single acquisition that only takes 0.8 s leads to a good quality CARS image (Fig. S11), with only a minor reduction of the signal-to-noise ratio compared to averaging over five acquisitions, as used for Fig. 5a.

Figure 5b demonstrates the SHG imaging performance for collagen fibers from a rat tail tendon. The close-up view of the fibril cross section (Fig. 5c) confirms the 0.8 µm two-photon lateral resolution of the flexible nonlinear endoscope. Lastly, we merge the CARS and SHG images into multimodal images in Fig. 5d and e. The samples are fresh tissues from human colon obtained directly from surgery without any staining. The images presented in Fig. 5d and e are obtained ~50 µm below the sample surface. The CARS images reveal the lipid content, whereas the SHG images are sensitive to the presence of collagen. The merged images reveal the complex morphological structure of the tissue, highlighting a clear separation between zones enriched in lipids and collagen fibers. We verified that moving the fiber during acquisition does not affect image quality such that the distal head can be oriented at will in relation with the tissue sample. All of the images shown in Fig. 5 were obtained at room temperature and with a twofold twist of the fiber onto itself with a 7 cm radius of curvature. Finally we tested the operation of our flexible endoscope with the probe immersed in water. Although the beam focus is slightly enlarged (Fig. S12a), due to the longer focal length of the miniature objective, the CARS images are similar in terms of contrast and resolution (Fig. S12b). Altogether, these results demonstrate the ability of the fiber endoscope to perform multimodal label-free nonlinear imaging directly on fresh tissues with a submicron lateral resolution and ~8 μm axial resolution (Fig. S8), as shown by *z*-scanned SHG and CARS images (Fig. S13a, b).

The major challenge faced by coherent Raman endoscopy is to simultaneously achieve a high spatial resolution, large FoV and high CARS contrast. Earlier attempts using solid-core fibers have been limited by the strong FWM background that occurs inside the solid core^9–11^, which has a negative impact on the CARS image contrast quality and/or the bulkiness of the distal endoscope head^9,13,15^. Our double-clad Kagomé-lattice hollow-core fiber solves all of these issues, enabling distortion-less delivery of femtosecond excitation pulses^24^ that generate strong signals from the samples, which are efficiently back-collected by the fiber. When combined with the microsphere inserted in the distal core, the fiber shows a micron-sized distal focus (Fig. 3d, Fig. S9) enabling a large FoV operation—due to the close to unity magnification of the four element miniature objective (Fig. S1). The low GVD of the HC fiber enables direct delivery of sub 200 fs pulses, avoiding the complexity associated with dispersion pre-compensation schemes^25^. Finally, the Kagomé lattice HC design provides a broad transmission window^26^ that is suitable for CARS to address high-frequency ~3000 cm^−1^ vibrational bonds found in lipids. This is in sharp contrast with earlier work performed by our group^27^ using HC band gap fibers with a narrow transmission window, that limits the detection of Raman shifts to wavenumbers below 1000 cm^−1^. Although femtosecond pulses provide strong signals that enable fast, highly contrasted CARS imaging of lipid structures (Fig. 5, Fig. S11), the low vibrational spectral resolution due to the broad spectra is a downside. To perform spectrally resolved CARS, picosecond pulses must be used. With longer pulse durations, strategies to remove the possible FWM background generated by the bead, naturally suppressed in femtosecond CARS (Fig. S6), might be considered.

With an external diameter of 4.2 mm, our endoscope distal head is small enough to be inserted into the user channel of many conventional endoscopes. Further improvements could be made in terms of miniaturization as smaller piezo-tubes can be used to reduce both the rigid length and the external diameter of the probe. Recently, a 2 mm outer diameter and 10 frames per second acquisition speed have been achieved using a similar resonant piezo scanning technology^28^. Our HC-based endoscope is also geared for future SRS imaging developments owing to the fact that parasitic SRS noise in a HC Kagomé lattice fiber is lower by a factor of 10^4^–10^6^ compared to a solid-core fiber and can even be completely suppressed^29^.

## Conclusions

We reported a fiber endoscope for high-resolution multimodal nonlinear imaging by merging several innovations: a hollow-core fiber with a Kagomé lattice, dual fiber cladding, microsphere focusing, and resonant miniature piezo scanner. This unique combination of key technologies solves most of the issues raised in multimodal nonlinear endoscopy: ultra-short pulse delivery, efficient signal collection, large field of view, submicron resolution, low noise and compactness. High-contrast and high-resolution CARS and SHG nonlinear endoscopy were achieved in unstained tissues with a submicron resolution over a FoV of up to 320 µm. This endoscopy platform is highly versatile and fully compatible with recent coherent Raman laser source developments^30,31^ as well as with any nonlinear imaging technique using wavelengths lying within the broad transmission window of the HC. This includes TPEF, SHG, THG, CARS, SRS, and also a broad variety of pump-probe schemes^32^. This endoscope platform opens up interesting perspectives for intraoperative label-free imaging for real-time histopathology diagnosis^5^.

## Electronic supplementary material

Supplementary Figure(DOCX 11171 kb)
