# Supplementary material for: High-resolution multimodal flexible coherent Raman endoscope
Source: Light Sci Appl. 2018 May 30;7:10. doi: 10.1038/s41377-018-0003-3 (PMC6107025; doi:10.1038/s41377-018-0003-3)
Supplement: Supplementary file 1 — Supplementary Figure(DOCX 11171 kb) [file 41377_2018_3_MOESM1_ESM.docx]

**Supplementary Information**

**High-resolution multimodal flexible coherent Raman endoscope**

**Alberto Lombardini^1^, Vasyl Mytskaniuk^1^, Siddharth Sivankutty^1^, Esben Ravn Andresen^1,2^, Xueqin Chen^1^, Jérôme Wenger^1^, Marc Fabert^3^, Nicolas Joly^4^, Frédéric Louradour^3^, Alexandre Kudlinski^2^, and Hervé Rigneault^1*^**

^1^ Aix Marseille Univ, CNRS, Centrale Marseille, Institut Fresnel, Marseille, France

^2^ Université Lille, CNRS, UMR 8523, Laboratoire de Physique des Lasers Atomes et Molécules, F-59000 Lille, France

^3^ Université de Limoges, CNRS, XLIM, UMR 7252, Université de Limoges, F-87060 Limoges, France

^4^ Max Planck Institute for the Science of Light, and Department of Physics, University of Erlangen Nuremberg, 91058 Erlangen, Germany

[^*^herve.rigneault@fresnel.fr](mailto:*herve.rigneault@fresnel.fr)

**CONTENT**

0- **Details on the experimental setup**

1- **Figure S1**: Miniature objective lens

2- **Figure** **S2**: Resonant four-quartered piezo scanner

3- **Figure** **S3**: Resonant piezo scanner calibration and image acquisition

4- **Figure** **S4**: Image reconstruction

5- **Figure** **S5**: HC fibers deliver fs pulses without time and spectral distortions

6- **Figure S6**: HC fiber enables background-free CARS detection

7- **Figure S7**: The HC double clad collects the generated nonlinear signals in the case of scattering samples

8**- Figure S8:** CARS PSF estimation

9- **Figure S9**: PSF off-axis aberrations are negligible for a FoV up to 350 µm

10- **Figure S10**: Probe transmission imaging through USAF-1951 resolution chart.

11- **Figure S11**: Comparison between CARS averaged images and single acquisition

12- **Figure S12:** Imaging in liquid environment

13- **Figure S13:** Depth-resolved SHG and CARS imaging with the flexible nonlinear endoscope

**14- Figure S14:** Color bars and count rates for Fig. 4c and Fig. 5

**0- Details on the experimental setup**

Two synchronized 80 MHz femtosecond pulse trains (pump 800 nm – 100 fs, Stokes 1040 nm – 160 fs) are delivered by a fs laser system (Discovery, Coherent, Santa Clara, USA). The beam powers were controlled with half-waveplates and polarizing beamsplitters. The temporal delay between the pulses was adjusted by means of a retroreflector mounted on a mechanical translation stage (1 μm steps). The beams were spatially combined with a notch filter optimized for the reflection of the 1040 nm beam (NFD01-1040, Semrock, Rochester, USA). The beams were injected in the fiber with a 40x microscope objective (NA=0.6, LUCPLFLN 40x, Olympus, Shinjuku, Japan). The high NA of the lens allowed to collect the entire signal back-collected by the fiber double clad (NA=0.5 at 400 nm). The use of a telescope (f_1_=400 mm, f_2_=40 mm) was necessary to reduce the diameter of the excitation beams before injection in the fiber core owing to its low (0.02) numerical aperture. The excitation beams propagated in 1 m of the Kagomé DC fiber, whose distal end was attached to the four-quartered piezo-tube (PT230.94, PI Ceramic, Karlsruhe, Germany). The silica micro-bead (9000 Series Glass Particle Standards, 30 μm, Thermo Scientific, Waltham, Massachusetts, USA) attached to the fiber core focuses the beams to a micron sized spot. This spot is re-imaged on the sample by means of a 4-lens (Edmund optics) miniature objective described in the supplementary section (Fig. S1). The nonlinear signals generated by the sample were back-collected by the miniature objective and coupled in the Kagomé DC fiber, to be detected on the endoscope proximal side. A long-pass dichroic beamsplitter (FF757-Di01, Semrock, Rochester, USA) was used to separate the detection path (<750 nm) from the excitation path (>770 nm). The detection path was composed of the coupling objective lens (NA=0.6, LUCPLFLN 40x, Olympus - f=4.5 mm), a telescope lens (f=40 mm), a 100 mm lens and a 50 mm tube lens. Bandpass filters placed in front of the photomultiplier tube (H7421-40, Hamamatsu Photonics, Japan) allowed to separate the photons generated by each nonlinear process (TPEF, CARS, SHG).

For calibration purpose only, a microscope objective (LUCPLFLN 40x, Olympus) placed in front of the endoscope distal head allows (i) to measure the spot position during a scan with a position sensitive detector (PDP90A, Thorlabs, Newton, New Jersey, USA) and (ii) to image the distal end of the fiber on a CCD camera (DCC1545, Thorlabs).

The resonant fiber-scan and signal detection were controlled by a 1 MHz data acquisition board (NI USB-6151, National Instruments, Austin, Texas, USA). Custom LabVIEW software synchronized the detection and scan tasks by means of a start trigger and the internal clock of the board (details of the synchronization algorithm can be found in). The images were then reconstructed by means of an open-loop algorithm (described in the supplementary section Fig. S4), that displayed the nonlinear images in real-time in the user-friendly scan-software interface[^1^](#_ENREF_1).

The samples used for nonlinear image calibration in Fig. 4 consisted of fluorescent beads (Thermo Fisher Scientific, Carboxylate-Modified Microspheres, 0.2 µm, yellow-green fluorescent (505/515)) and polystyrene beads (79633-5ML-F, St. Louis, Missouri, USA).

The human colon tissues were sandwiched between two coverslip and imaged with no further staining or preparation. The images presented on Fig. 5 were obtained with the endoscope distal head rigidly mounted on 3D translation stage.

**1. Miniature objective lens**

**
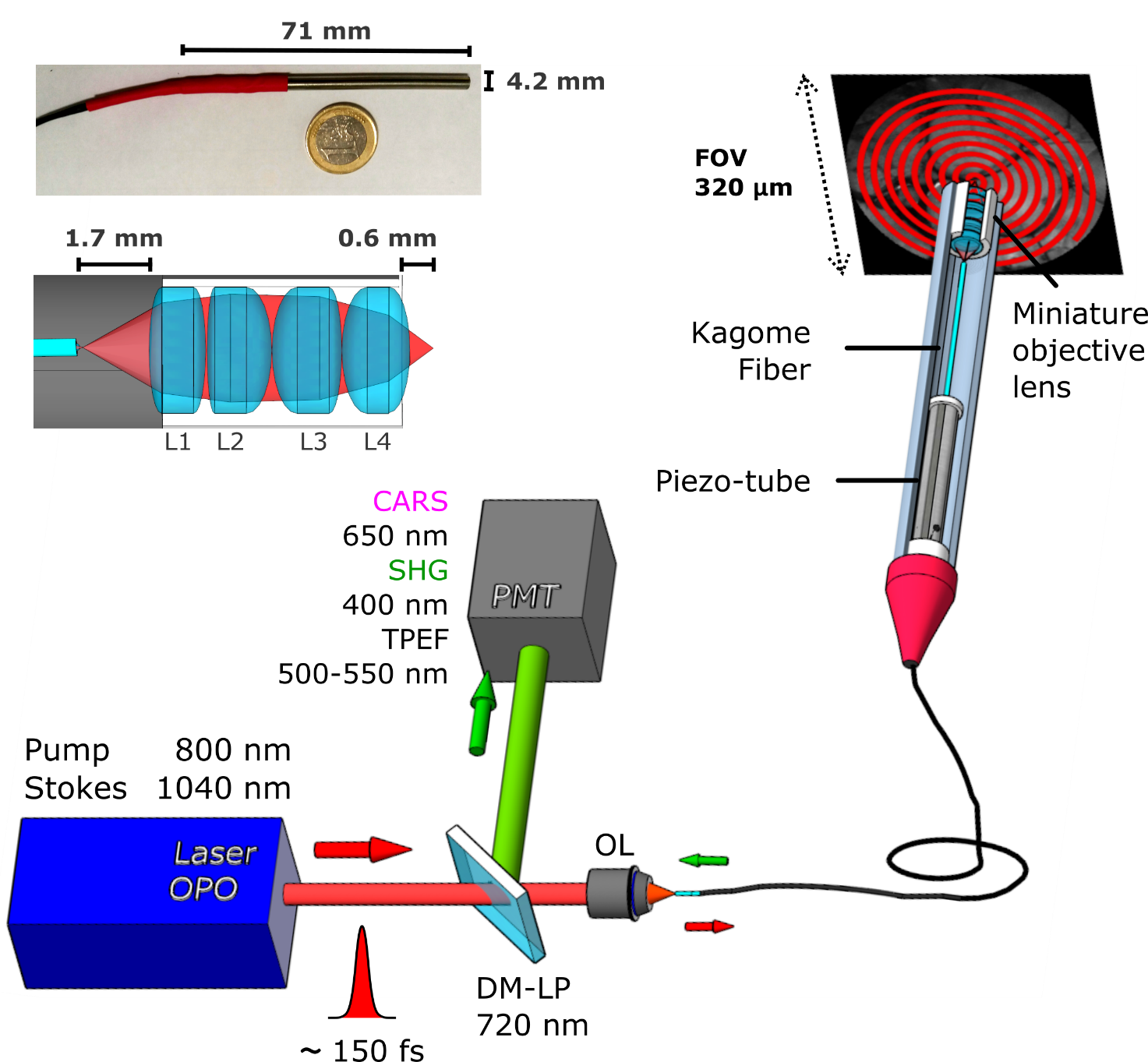
**

**Figure S1:** Detailed view of the endoscope distal head featuring the miniature objective lens made of four 2 mm diameter achromatic doublets providing a 0.63 magnification. The focal lengths for the lenses are L1: f=9 mm, L2: f=6 mm, L3: f=4 mm, L4: f=3 mm. Each lens has a thickness of 2 mm. Reference of the Edmund Optics (EO) microlenses: L1 - EO #83-338, L2 – EO #65-569, L3 – EO #65-568, L4 – EO #65-567.

**2. Resonant four-quartered piezo scanner**


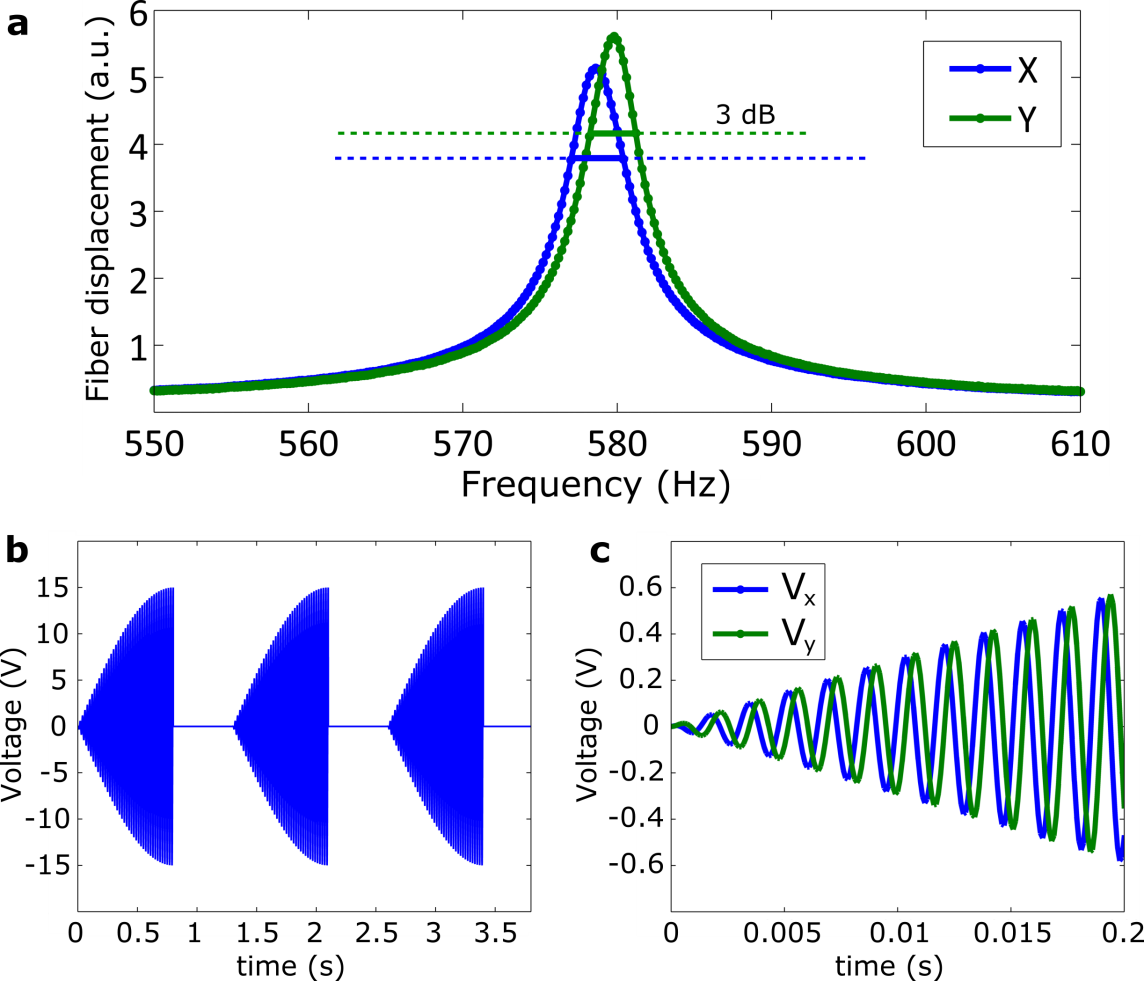


**Figure S2:** (a) Resonant frequency of the piezo X and Y axis with a 23 mm fiber free standing length. (b) Piezo driving voltage as a function of time, the image acquisition is performed during the expanding pattern and takes 0.8 s, which is followed by a 0.5 s rest period; (c) voltages applied along the X (V*_x_*) and Y (V*_y_*) piezo axis during the first 0.2 s of the expanding pattern showing. The π/2 phase shift between V*_x_* and V*_y_* leads to the spiral scan.

3**. Resonant piezo scanner calibration and image acquisition**


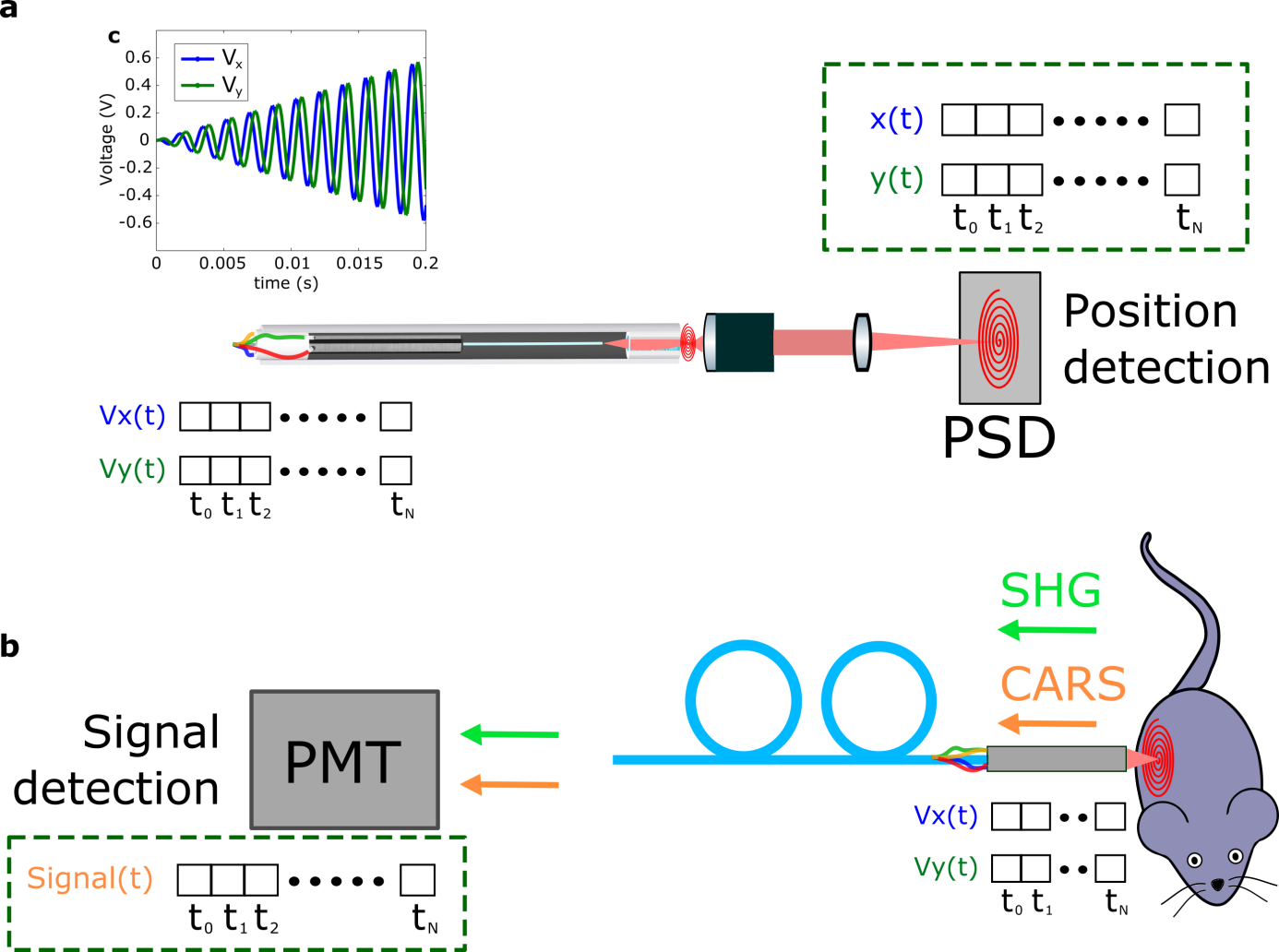


**Figure S3:** Imaging with the fiber scanner requires the measurement of a signal and the knowledge of the instantaneous spot position. The latter is obtained in a first calibration step (a), where the beam position is tracked during a scan with a position sensitive detector (PSD - PDP90A, Thorlabs). When imaging is performed (b), the same waveforms V_x_(t) and V*_y_* (t) are applied to the piezo-tube and the nonlinear signals (CARS, SHG, TPEF) are back-collected by the endoscope distal probe, transmitted by the fiber to the proximal photomultiplier tube (PMT - H7421-40, Hamamatsu) that records the signal S(t).

The positions and signal vectors are then used by the software to reconstruct the images, as shown in **Fig. S4**. A daily calibration with the PSD is used to compensate for small, probably thermal-induced drifts in the scan pattern.

**4. Image reconstruction**


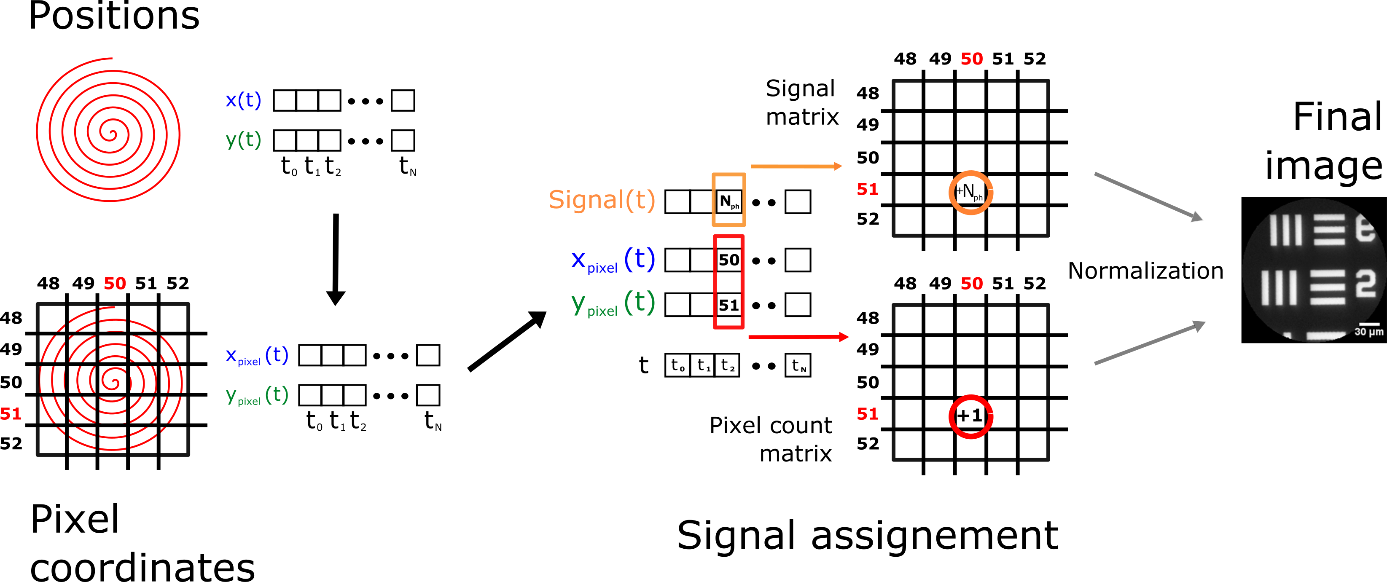


**Figure S4:** The image reconstruction step is performed right after the signal acquisition from a scan. First of all, the spot positions measured in the calibration step (x(t) and y(t), see **Fig. S3**) are mapped into discrete pixel coordinates (x_pixel_(t) and y_pixel_(t)). These coordinates correspond to the elements of a square matrix whose size is equal to the desired image size (arbitrarily defined by the user). The image is then built from the simultaneous readout of the signal vector and the pixel coordinate vectors. The intensity of the signal detected at each specific time during the scan is assigned to the pixel in the image that corresponds to the spot position at that same time. Since the sampling of the FoV over time is not uniform, a matrix (pixel count) has to be built that contains the number of times that each pixel is sampled. This matrix is then used to normalize the one that contains the signal information. This normalization results in the desired image. Fiber driving and signal acquisition are synchronized and controlled with custom designed LabVIEW software.

**5. HC fibers deliver fs pulses without time and spectral distortions**

**
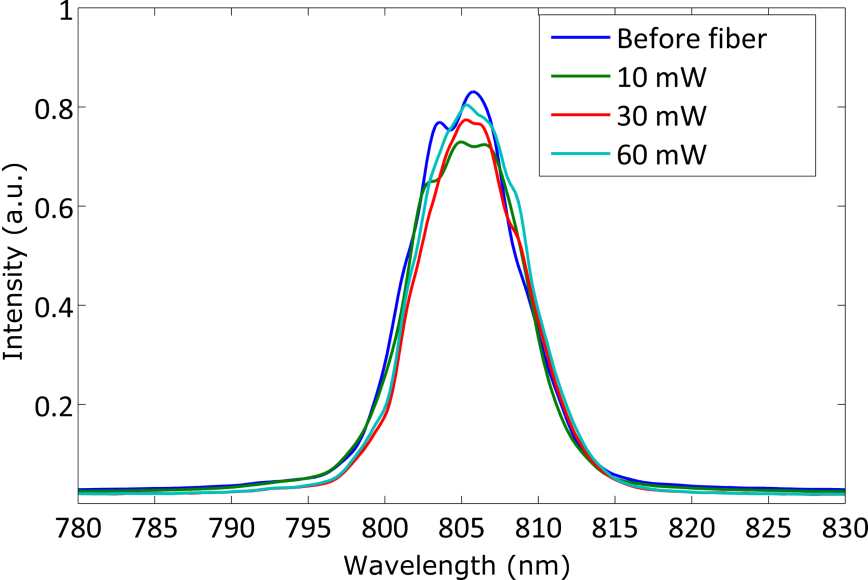
**

**Figure S5:** Pulse spectrum measurements before and after 1 m of propagation in the HC fiber for different input powers. The weak air filled core nonlinearity does not affect the fs pulse spectrum for the considered power levels.

**6. HC fiber enables background-free CARS detection**


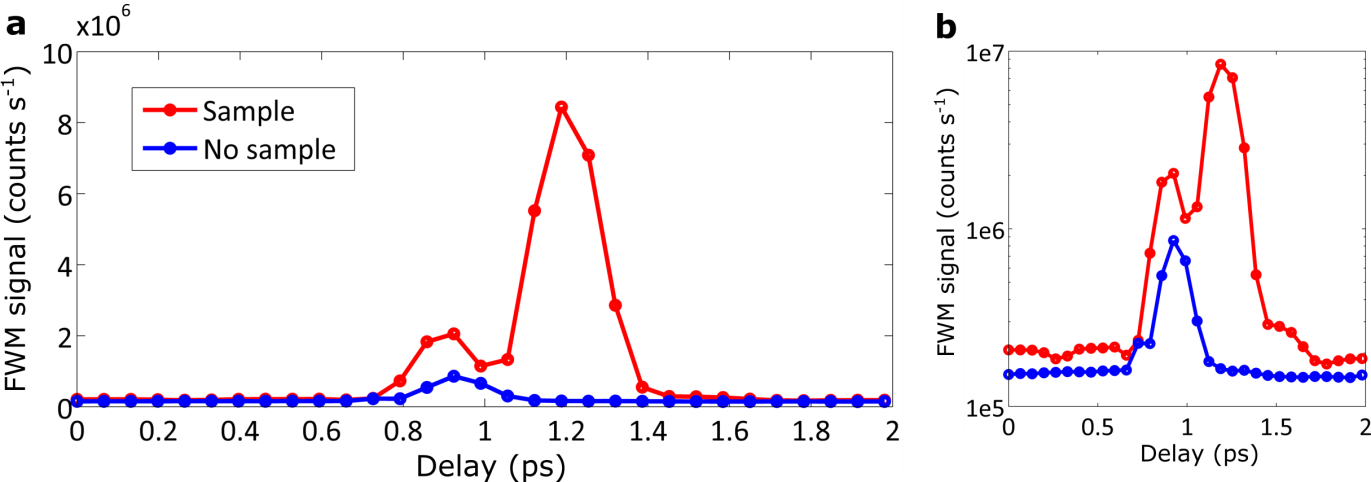


**Figure S6**: CARS signal epi-detected by the flexible endoscope probe when scanning the delay between the pump and the Stokes pulses. In the absence of the sample (blue curve), a weak FWM background is found at 0.9 ps delay between the pump and Stokes pulses. We attribute this background to the silica microsphere lens inserted into the fiber core. In the presence of the olive oil sample (red curve), this background is still present but it is clearly separated from the strong CARS signal from the lipids arising at 1.2 ps delay. This delay difference is attributed to the inherent dispersion of the miniature objective. Importantly, the background without the sample is vanishing for the 1.2 ps delay leading to optimum pump and Stokes pulses overlap in the sample plane. (b) same image as (a) in log scale.

**7. The HC double clad collects the generated nonlinear signals in the case of scattering samples**


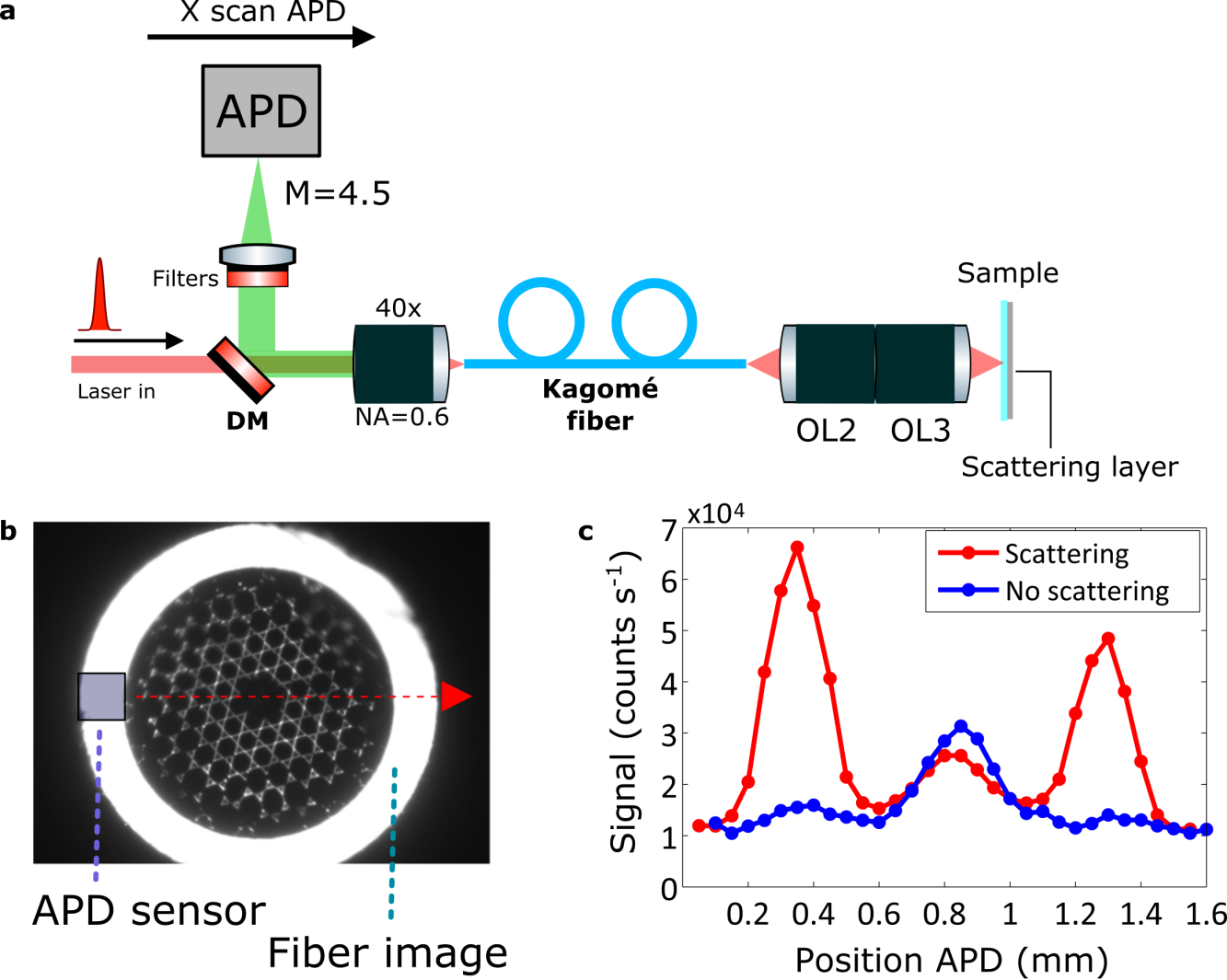


**Figure S7:** (a) setup of the experiment. The sample is a fluorescein (FITC) cuvette solution excited at 900 nm. The TEPF signal collected by the HC fiber is filtered in the 500 – 550 nm band and analyzed spatially by scanning an APD with a small 150 µm active surface (SPCM, Perkin Elmer). For this, the output facet of the HC-DC fiber is magnified (x4.5) and imaged onto the APD; (b) output facet of the HC-DC fiber showing the de-magnified image of the APD active surface and the performed linear scan (red dash line). (c) Collected TPEF signal across the HC-DC output facet when the back surface of the FITC cuvette is made clear (blue) and when it is covered with a thin layer of TiO_2_ scattering nanoparticles (200 nm diameter – layer thickness 500 µm) (red). Most of the back emitted TPEF signal is collected by the DC in the case of a scattering sample whereas it is collected by the air core in the case of a non-scattering sample. All measurements are performed with a Chameleon (Coherent) Ti:Saph laser.

**8. CARS PSF estimation**


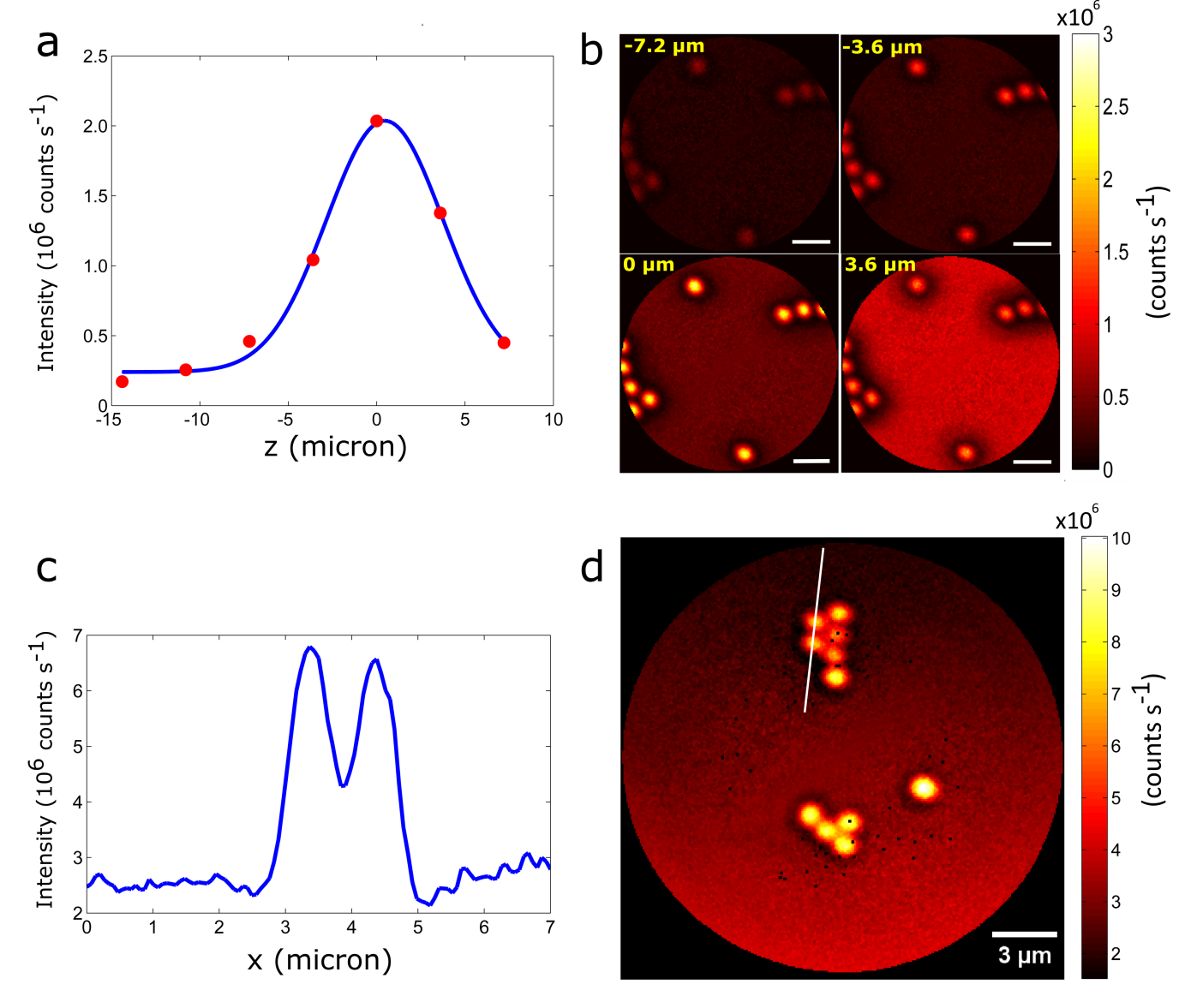

**Figure S8:** Estimation of the CARS point spread function. The axial resolution is measured as 7.7 µm (a) from the FWHM of the intensity profile of 2 micrometer beads imaged at different positions (-7.2 µm, -3.6 µm, 3.6 µm) with respect to the focal plane (0 µm) (b). The CARS signal was detected in the forward direction with excitation powers of 2 mW (Pump) and 1 mW (Stokes). The CARS images of individual 1 micron beads (d) provide an estimation of the CARS lateral PSF which is approximately 1 µm as shown by the intensity profile of two individual adjacent beads (c). Image (d) was acquired detecting the forward generated CARS signal. The polystyrene beads were deposited on a glass coverslip, which is responsible for the strong non-resonant CARS background in (d). The excitation powers were approximately 4 mW (Pump) and 2.5 mW (Stokes).

**9. PSF off-axis aberrations are negligible for a FoV up to 350** **µm**

**
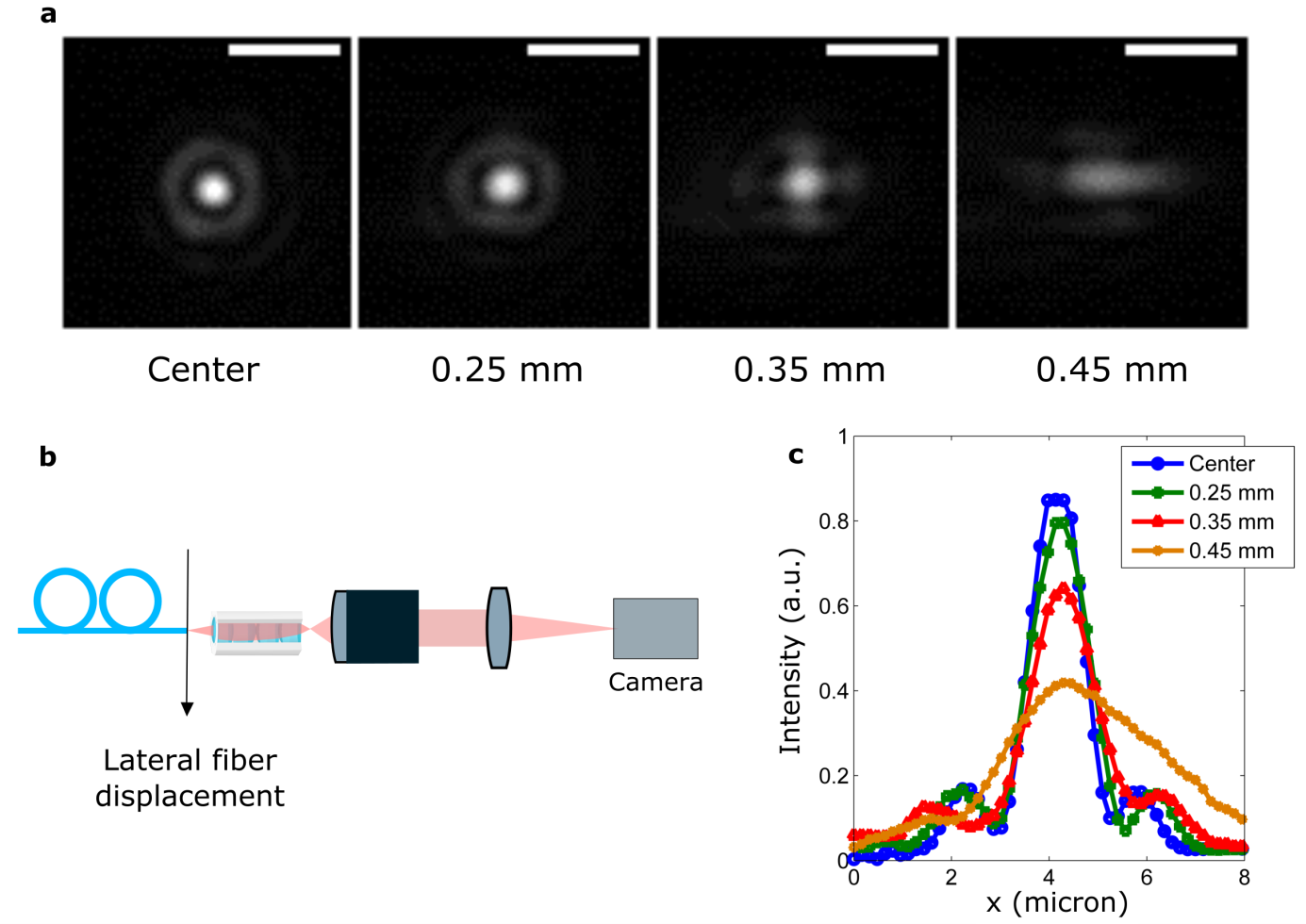
**

**Figure S9:** (a) Transmission PSF evolution when the HC-DC fiber distal facet is laterally displaced from the optical axis of the miniature objective (the magnification between the HC-DC distal facet and the CCD camera is 33), as shown in Fig. (b). The beam profile is not significantly affected, up to a lateral displacement of 250 µm (FoV 500 µm) (intensity profiles in (c)). However, when the lateral displacement reaches 350 µm the beam profile becomes slightly asymmetric, up to an elongated profile observed at 450 µm. We conclude that the probe has a uniform PSF across a FoV of 320 µm (corresponding to 500 µm piezo fiber scan - with a de-magnification of 0.63). All measurements are performed with a Chameleon (Coherent) Ti:Saph laser operating at 800 nm.

**10. Probe transmission imaging through USAF-1951 resolution chart.**


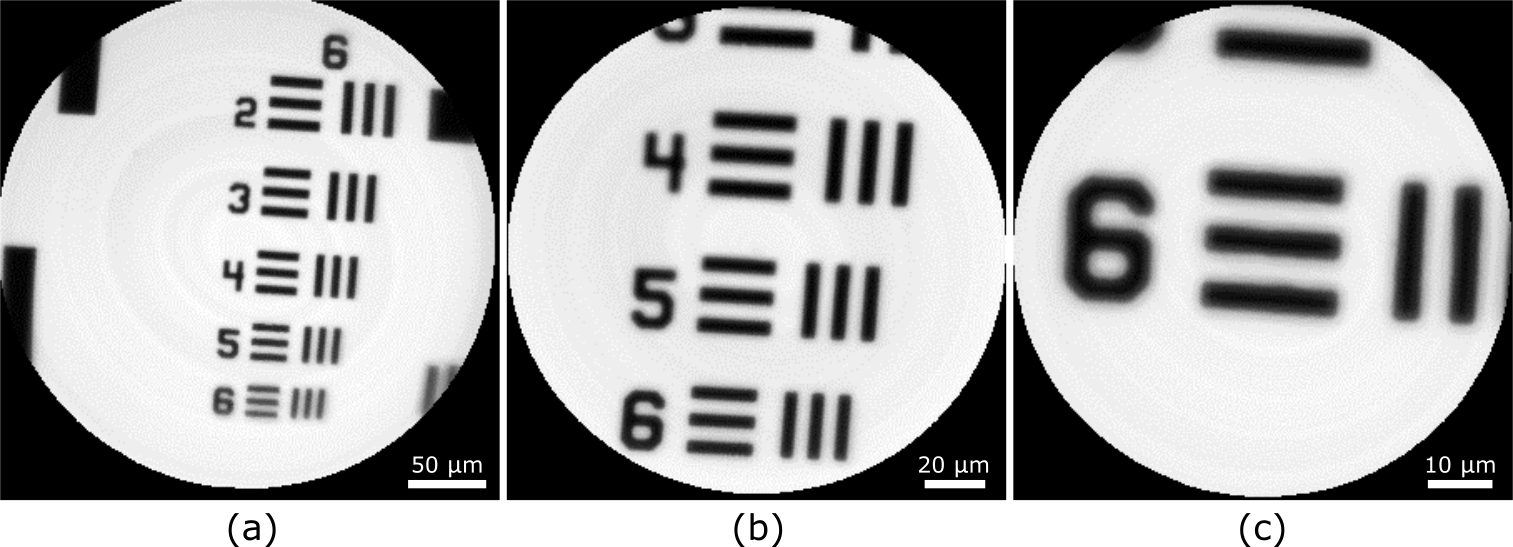


**Figure S10:** Probe transmission imaging through a USAF-1951 resolution chart with driving voltages ±15 V (30 V pk-pk) (a), ±7.5 V (b) and ±3.75 V (c). The experiments were performed by recording the transmitted signal with a photodiode (DET10A, Thorlabs) as the laser spot was scanned across the transmission chart.

**11. Comparison between CARS averaged images and single acquisition**


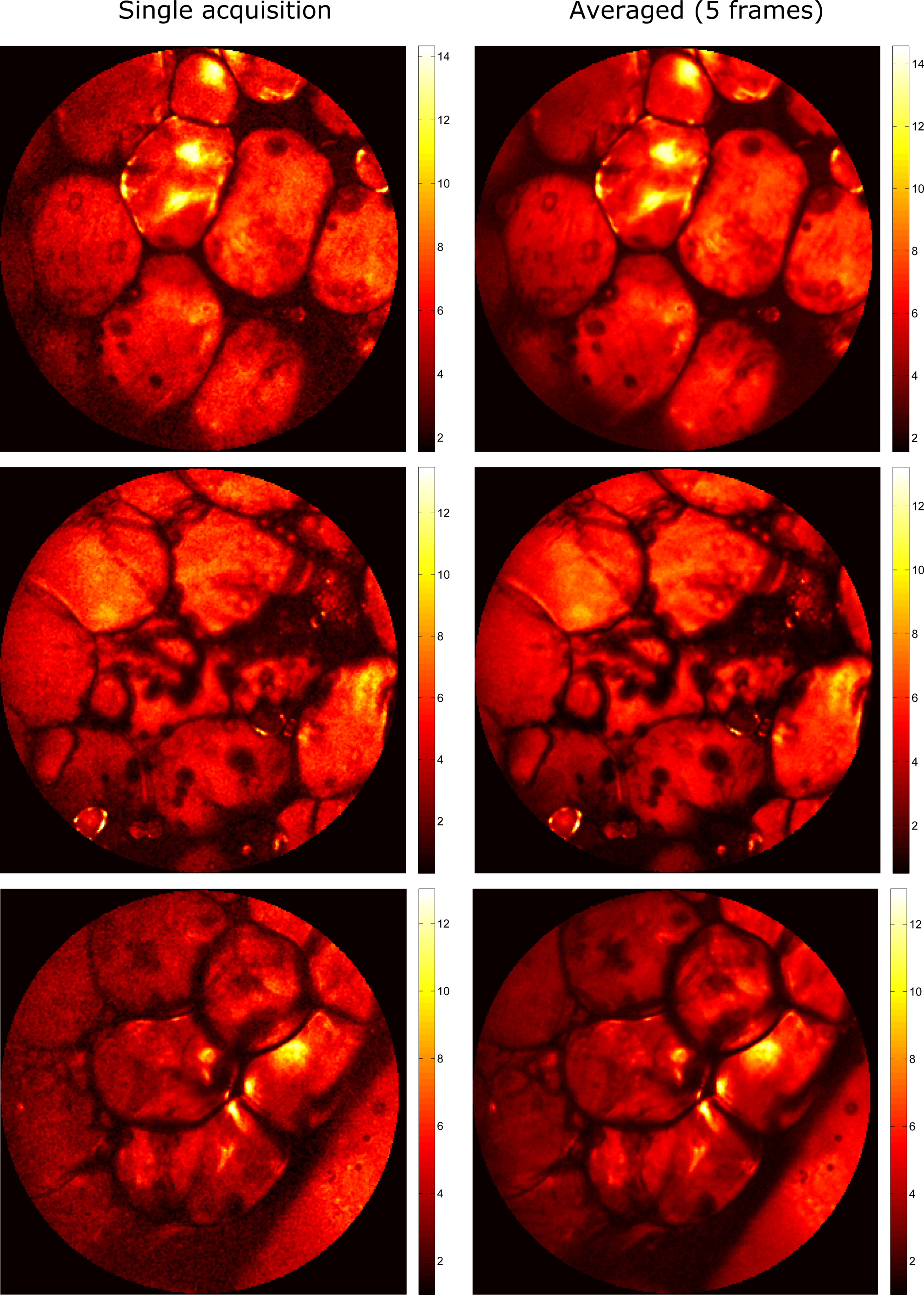


**Figure S11:** Comparison of epi detected CARS images from a human colon tissue with 5x averaging / 6.5 s acquisition time and a single 0.8 s acquisition. Color bar range is (10^6^ counts s^-1^) for all images. Power on the sample: P_pump_=20 mW, P_Stokes_=10 mW. All measurements are performed with a Discovery (Coherent) operating at 800 nm and 1040 nm. FOV=320 μm for all images.

**12. Imaging in liquid environment**

**
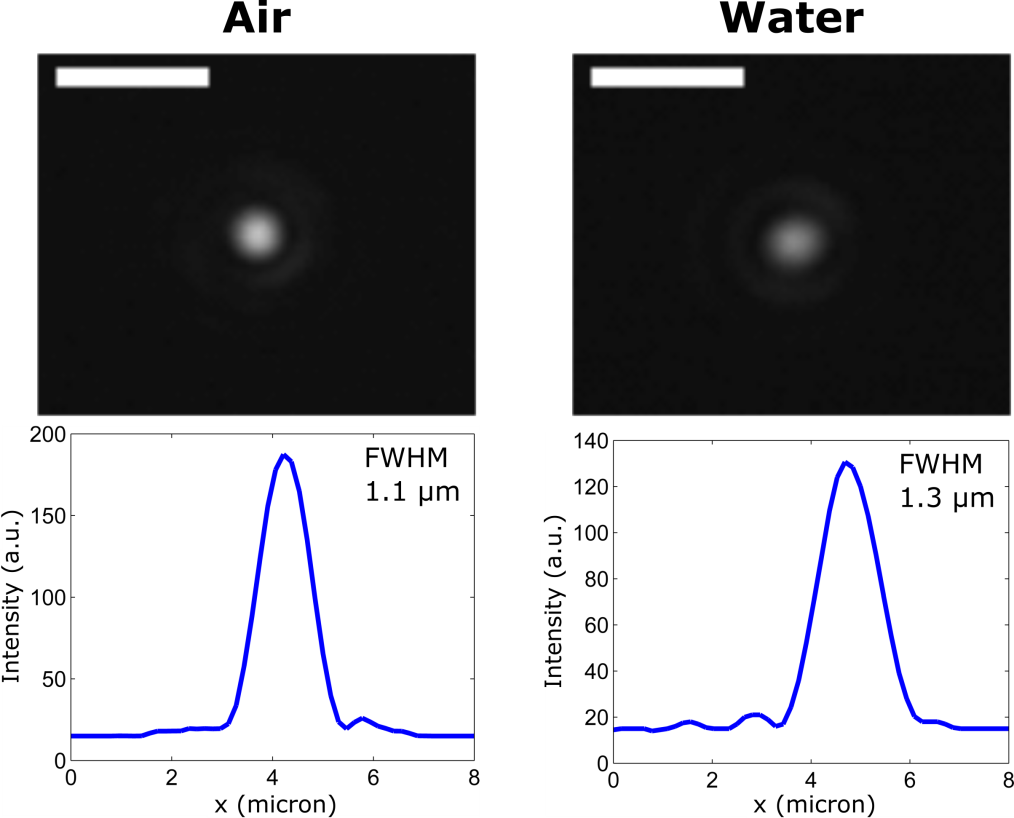
**

**Figure S12a:** Beam focus in the air and in water (800 nm beam). Scale bar is 5 µm.


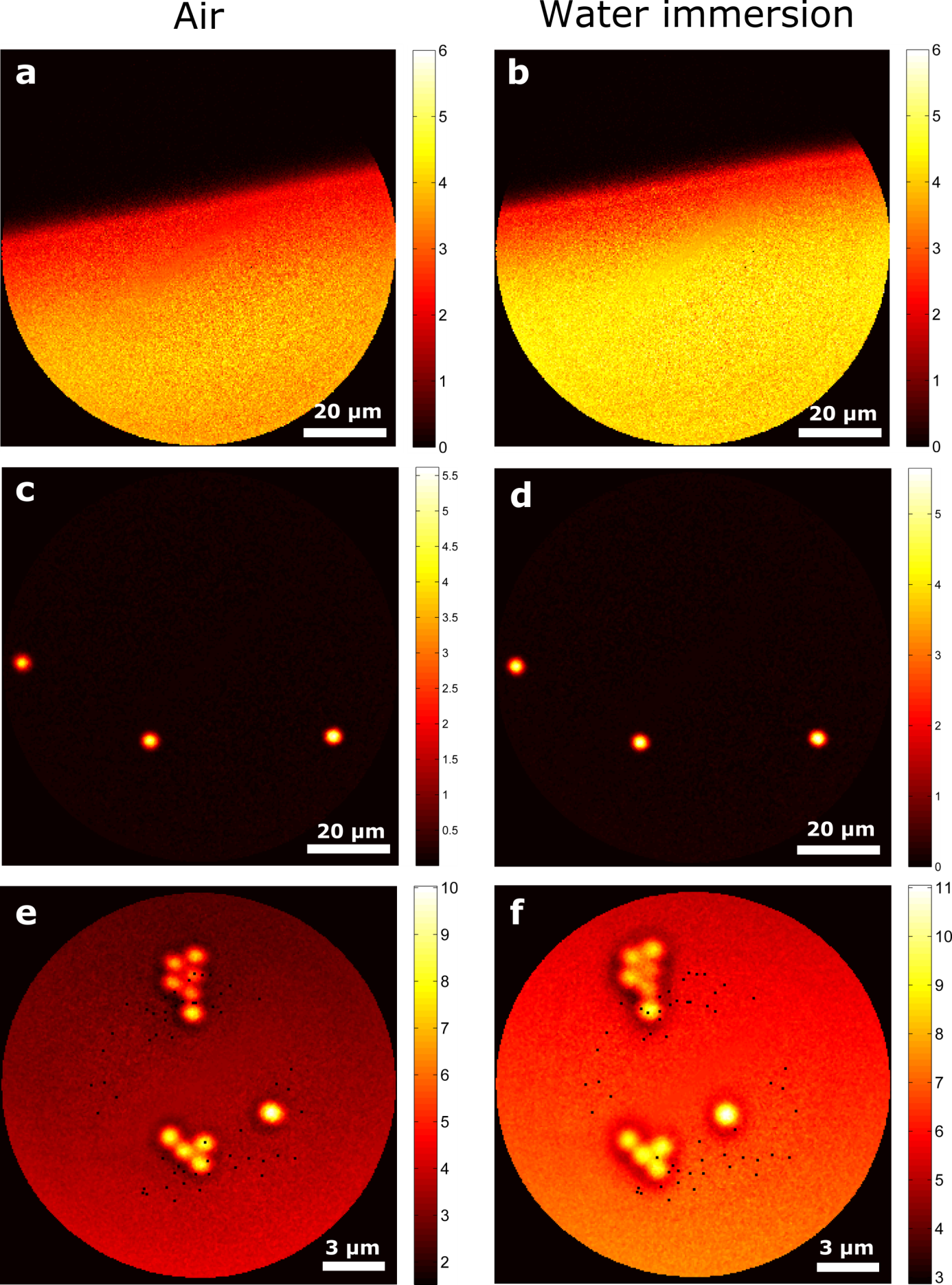


**Figure S12b:** Forward-detected CARS images of different samples, with the medium between the probe and the sample being air (left column) or water (right column). (a) and (b) interface between air and olive oil (pump: 1.5 mW, Stokes: 1 mW), (c) and (d) 5 µm polystyrene beads (pump: 2 mW, Stokes: 1.3 mW), (e) and (f) 1 µm polystyrene beads (pump: 4 mW, Stokes: 2.7 mW). Range is expressed in (10^6^ counts s^-1^).

**13. Depth-resolved SHG and CARS imaging with the flexible nonlinear endoscope**

**
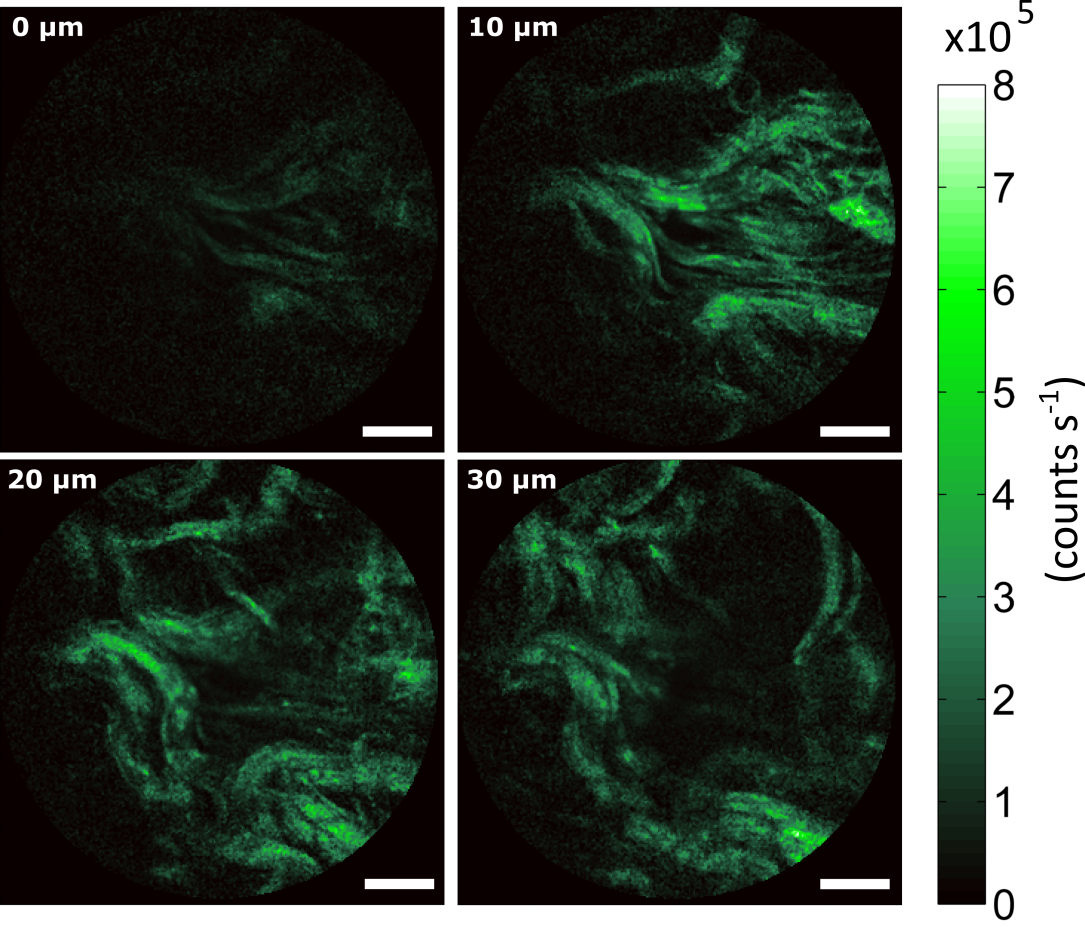
**

**Figure S13a**: Epi-detected SHG images of a human colon tissue ex-vivo. The images were acquired at different depths inside the tissue (as indicated in the top left corner of the image), the tissue section was moved with a translation stage. The 0 micron position does not necessarily represent the tissue surface, but the position of the first identified collagen structures. The scale bar is 50 micrometers for all images. The excitation power was 60 mW (800 nm excitation wavelength, 395-405 nm detection, images averages of 10 frames).


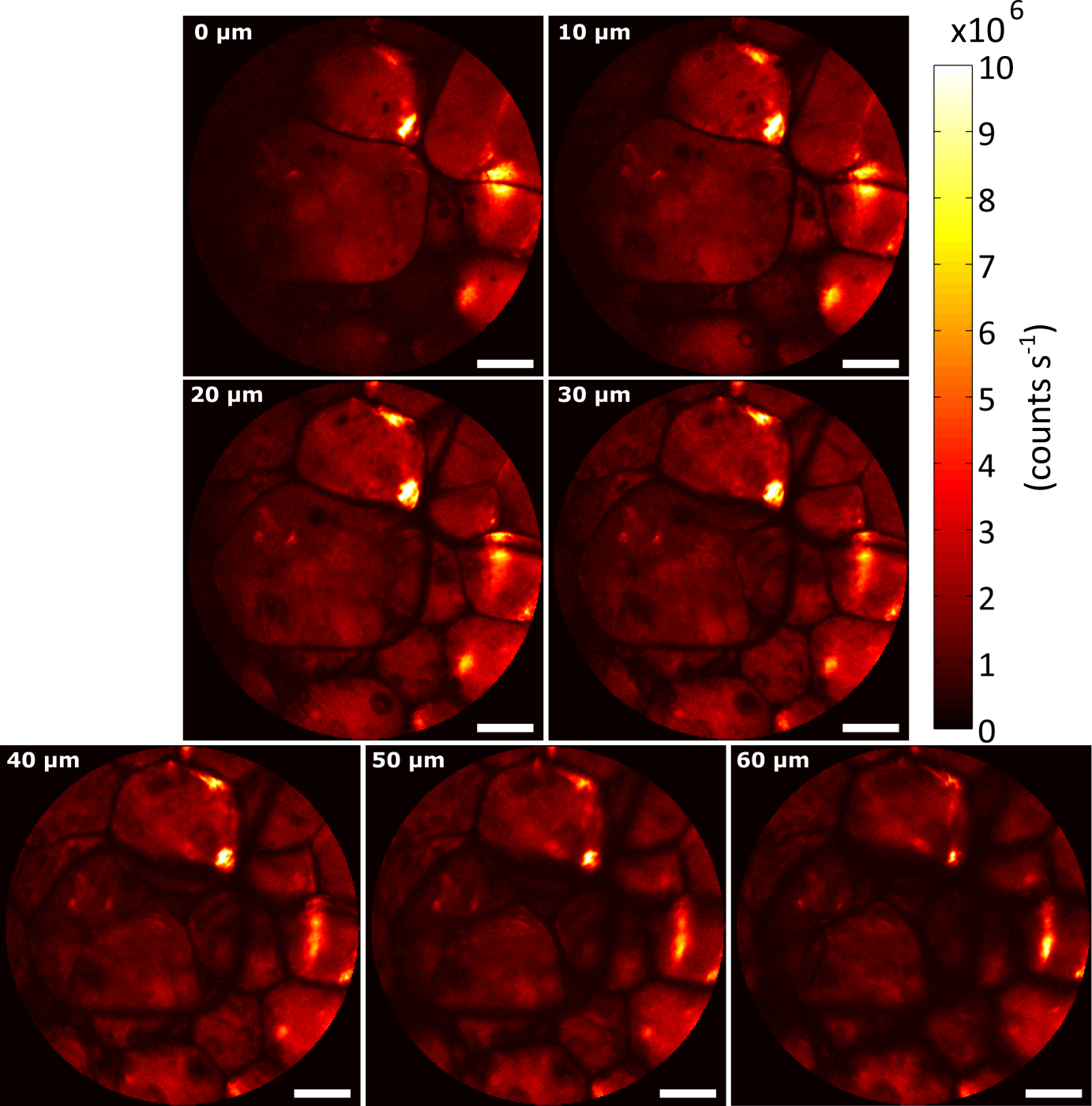


**Figure S13b:** Epi-detected CARS images of the fatty tissue in a human colon tissue ex-vivo. The images were acquired at different depths inside the tissue (as indicated in the top left corner of the image); the tissue section was moved with a translation stage. The 0 micron position represents the position most superficial imaged plane (estimated about 5 microns below the most superficial tissue surface point). The scale bar is 50 micrometers for all images. The excitation power was 45 mW (30 mW for the 800 nm pump beam, and 15 mW for the 1040 nm Stokes beam, images averages of 5 frames).

**14. Color bars and count rates for Fig. 4c and Fig. 5**


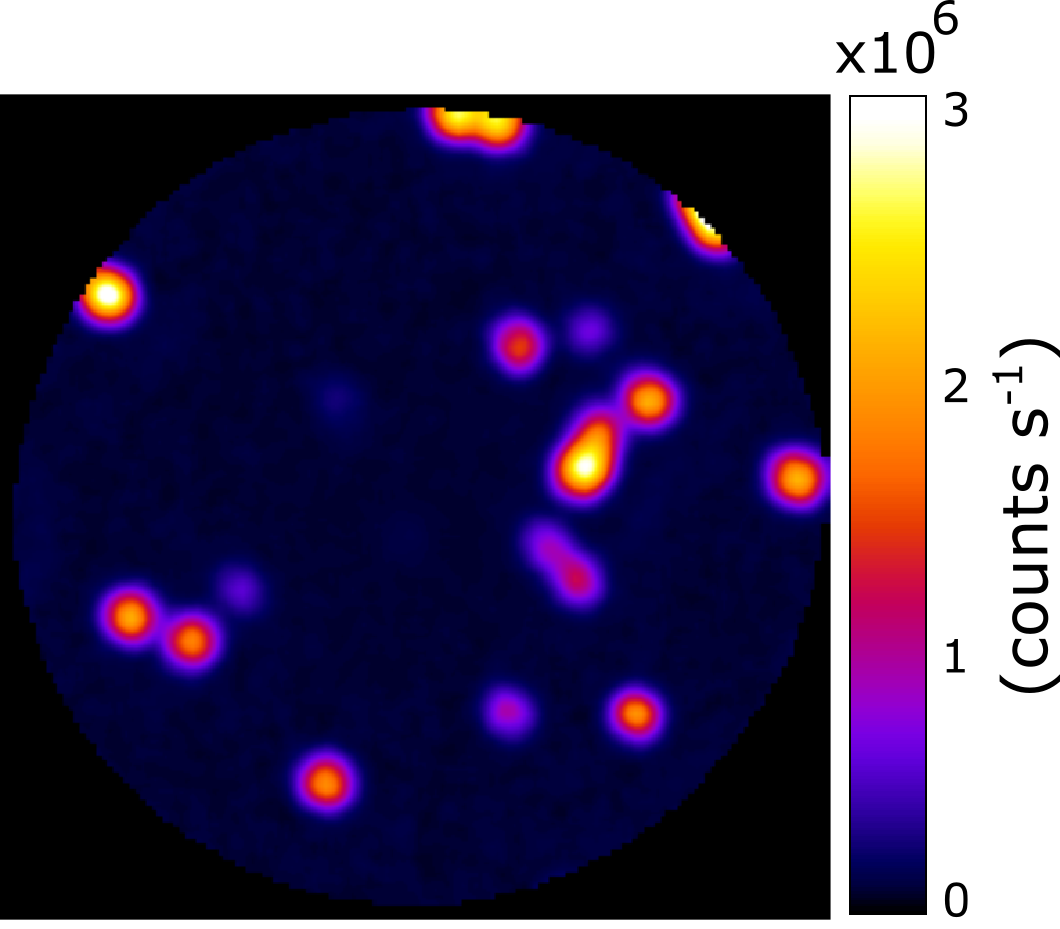


**Figure S14a:** Color scales and range of **Fig.4c**. Details on the image acquisitions are found in the caption of **Fig. 4**.


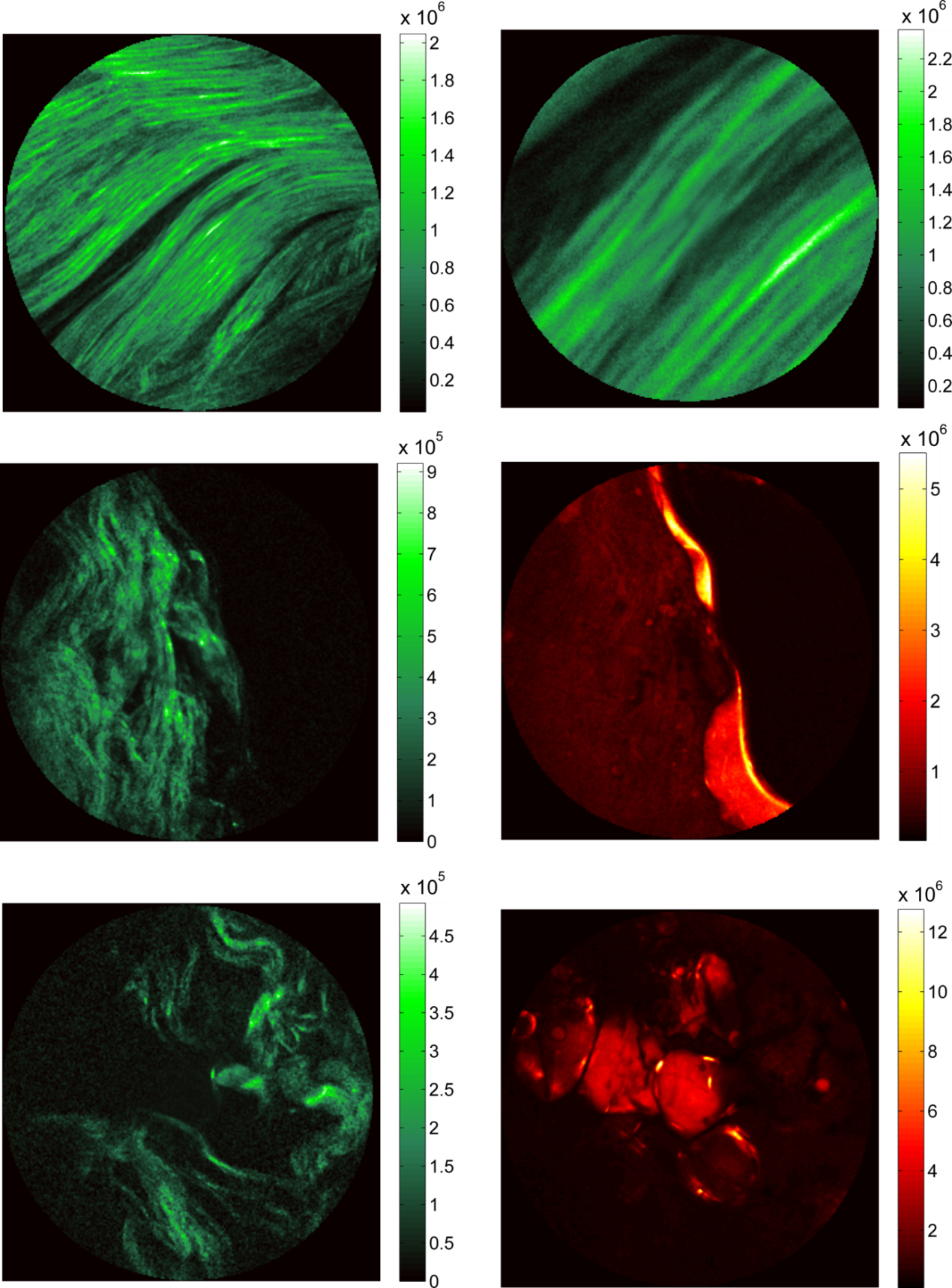


**Figure S14b:** Color scales and range (counts s^-1^) from **Fig.5**. Details on the image acquisitions are found in the caption of **Fig. 5**.

REFERENCE

1. Ferrand P. GPScan.VI: A general-purpose LabVIEW program for scanning imaging or any application requiring synchronous analog voltage generation and data acquisition. *Computer Physics Communications* 2015, **192:** 342-347.
